# The therapeutic value of bifidobacteria in cardiovascular disease

**DOI:** 10.1038/s41522-023-00448-7

**Published:** 2023-10-30

**Authors:** Jia Tang, Yumeng Wei, Chao Pi, Wenwu Zheng, Ying Zuo, Peng Shi, Jinglin Chen, Linjin Xiong, Tao Chen, Huiyang Liu, Qianjiao Zhao, Suyu Yin, Wei Ren, Peng Cao, Nan Zeng, Ling Zhao

**Affiliations:** 1 Key Laboratory of Medical Electrophysiology, Ministry of Education, School of Pharmacy of Southwest Medical University, Luzhou, 646000 P.R. China; 2 Luzhou Key Laboratory of Traditional Chinese Medicine for Chronic Diseases Jointly Built by Sichuan and Chongqing, The Affiliated Traditional Chinese Medicine Hospital of Southwest Medical University, Luzhou, Sichuan 646000 P.R. China; 3 Chengdu University of Traditional Chinese Medicine State Key Laboratory of Southwestern Chinese Medicine Resources, 1166 Liutai Avenue, Wenjiang District, Chengdu, Sichuan 611137 P.R. China; 4 Key Laboratory of Medical Electrophysiology, Ministry of Education, Development Planning Department of Southwest Medical University, Luzhou, Sichuan 646000 P.R. China; 5 Central Nervous System Drug Key Laboratory of Sichuan Province, School of Pharmacy of Southwest Medical University, Luzhou, Sichuan 646000 P.R. China; 6 Department of Cardiology, The Affiliated Hospital of Southwest Medical University, Luzhou, Sichuan 646000 P.R. China; 7 Department of Comprehensive Medicine, The Affiliated Traditional Chinese Medicine Hospital of Southwest Medical University, Luzhou, Sichuan 646000 P.R. China; 8 National Traditional Chinese Medicine Clinical Research Base and Drug Research Center of the Affiliated Traditional Chinese Medicine Hospital of Southwest Medical University, Luzhou, Sichuan 646000 P.R. China; 9 The Affiliated Hospital of Traditional Chinese and Western Medicine Nanjing University of Chinese Medicine, Nanjing, Jiangsu 210028 P.R. China

**Keywords:** Microbiome, Applied microbiology, Bacteria

## Abstract

There has been an increase in cardiovascular morbidity and mortality over the past few decades, making cardiovascular disease (CVD) the leading cause of death worldwide. However, the pathogenesis of CVD is multi-factorial, complex, and not fully understood. The gut microbiome has long been recognized to play a critical role in maintaining the physiological and metabolic health of the host. Recent scientific advances have provided evidence that alterations in the gut microbiome and its metabolites have a profound influence on the development and progression of CVD. Among the trillions of microorganisms in the gut, bifidobacteria, which, interestingly, were found through the literature to play a key role not only in regulating gut microbiota function and metabolism, but also in reducing classical risk factors for CVD (e.g., obesity, hyperlipidemia, diabetes) by suppressing oxidative stress, improving immunomodulation, and correcting lipid, glucose, and cholesterol metabolism. This review explores the direct and indirect effects of bifidobacteria on the development of CVD and highlights its potential therapeutic value in hypertension, atherosclerosis, myocardial infarction, and heart failure. By describing the key role of *Bifidobacterium* in the link between gut microbiology and CVD, we aim to provide a theoretical basis for improving the subsequent clinical applications of *Bifidobacterium* and for the development of *Bifidobacterium* nutritional products.

## Introduction

Cardiovascular disease (CVD) is the leading cause of death worldwide, accounting for ~30% of global mortality^1^. According to the World Health Organization (WHO), the global death toll from CVD was 17.9 million in 2021^2^. Physiological factors such as age, gender, and genetics as well as poor daily behavior are major CVD factors^3^. In addition, pathological states such as inflammation and oxidative stress and underlying diseases such as obesity, hyperlipidaemia (HLP), as well as diabetes have been proven to cause or exacerbate CVD^3,4^. However, a growing body of data has shown a correlation between intestinal microbiota and CVD, and that such microbiota hold great promise as an emerging therapeutic tool^5^. It has been reported that homeostasis of the intestinal microbiota, its physiological functions, and metabolites modulate human physiopathological processes and influence the developmental of CVD^6^.

*Bifidobacterium* belongs to the phylum Actinobacteria, a genus of high G + C Gram-positive, non-motile, rod-shaped, strictly anaerobic bacteria that are widely found in the intestinal tract and other luminal tissues of humans and animals^7–9^. As one of the first microorganisms to colonize the human gut and to accompany humans throughout their lives, bifidobacteria have a wide range of benefits for human health^8^. Bifidobacteria are one of the most important bacterial groups in the neonatal gut which promote proper development of physiological functions in neonates. In the early stages of life, bifidobacteria comprise up to 90% of the infant gut microbiota^8,10^. In the gut of adults, bifidobacteria make up 2–14% of the total gut microbial community; in the elderly, this number shrinks once again^11^. As probiotics, bifidobacteria are closely associated with human health and are best known for their role in promoting the health of the immune, digestive, and metabolic systems^8^.

Recent advances in research have shown that in the gut, which is rich in more than 1000 microorganisms, bifidobacteria show a significant potential for improving cardiovascular disease^12^. Interestingly, the abundance of bifidobacteria tended to decrease when cardiovascular events occurred, and conversely, their abundance was upregulated when CVD was ameliorated^13–15^. Emerging scientific research evidence suggested that bifidobacteria not only has a mitigating effect on inflammation, oxidative stress, but also improves intestinal barrier function as well as regulates intestinal microbial metabolites^14,16–18^. Furthermore, researchers have shown that obesity, HLP, and type 2 diabetes mellitus (T2DM), which are serious risk factors for CVD, could indirectly be reduced by bifidobacteria^19–21^. This review has mainly discussed the direct and indirect mechanisms of bifidobacteria in inhibiting the development of CVD, including suppression of oxidative stress, immunomodulation, and correction of lipid, glucose, and cholesterol metabolism to reduce classical risk factors for CVD. In addition, current advances in the use of bifidobacteria in hypertension (HP), atherosclerosis (AS), myocardial infarction (MI), and heart failure (HF) are discussed with an outlook on the future use of bifidobacteria as a cutting-edge therapeutic strategy for the prevention or treatment of CVD.

## Direct improvement of cardiovascular disease

### Antioxidant properties

Oxidative stress exists in various CVDs, such as AS, HP, myocardial ischemia-reperfusion (I/R) injury and HF, and strongly plays a role in its development or exacerbation^22^. The oxidative stress process occurs when there is production of excess reactive oxygen species (ROS), which include superoxide radicals, peroxyl radicals, hydroxyl radicals and hydrogen peroxide^23^. In AS, oxidative stress first causes endothelial dysfunction, thus increasing vascular permeability^24^. The oxidative stress then modifies the low-density lipoprotein (LDL) recruited to the artery walls into oxidized low-density lipoprotein (ox-LDL), which forms the basis for foam cells^24^. Finally, the increased ROS exacerbates plaque development^24^. On the other hand, ROS acts on vascular endothelial cell phosphorylation pathways and gene expression factors in HP to cause abnormal vascular tone. Myocardial I/R manifests as restoration of blood flow to hypoxic organs, a process that leads to excessive ROS production^25^. In addition, all phases of HF are accompanied by an excessive production of ROS^24^. This demonstrates that oxidative stress on the heart aggravates the prevailing CVD.

**Fig. 1:**
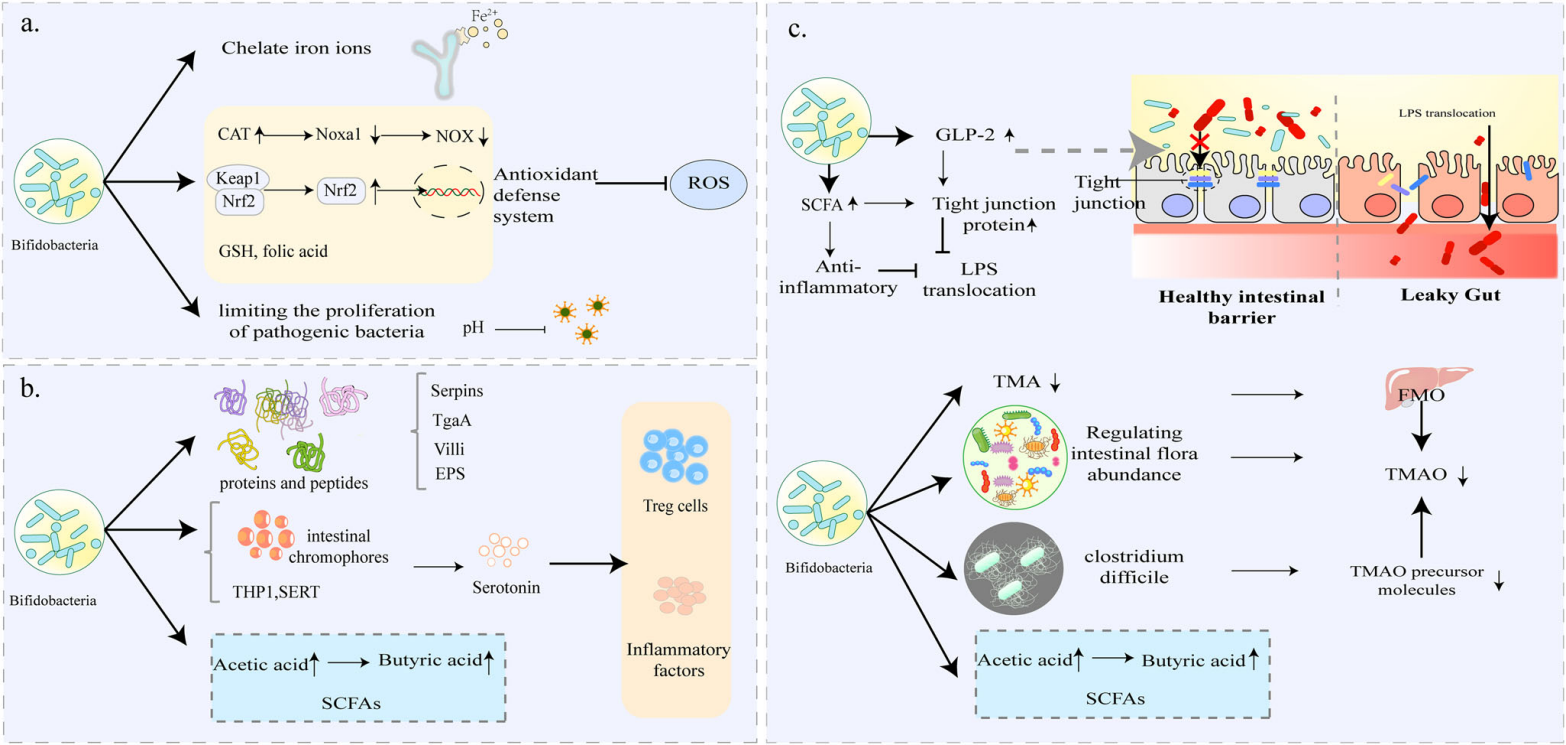
Direct Improvement of CVD. **a** *Bifidobacterium* may function as an iron ion chelator to inhibit oxidation and oxidation reactions. It can activate the key genes in the antioxidant defense system, produce glutamic acid (GSH) to maintain the cellular redox state, and inhibit the production of pathogenic bacteria through the acidic environment. **b** *Bifidobacterium* has special molecular structures such as Serpins, villi, TgaA, and EPS that allow it to participate in specific immunomodulatory processes and cause anti-inflammatory activity. Its metabolite acetate can also stimulate the production of butyrate, which has anti-inflammatory effects, by cross-feeding as a substrate. **c** *Bifidobacterium* can act on intestinal epithelial cell junctions to maintain the integrity of the intestinal barrier and inhibit LPS translocation; it can also protect the intestinal barrier from damage by controlling the production of TMAO, SCFAs and maintaining the abundance of intestinal microbial species. (The figure does not contain any third-party material, the figure and each of the elements in the figure were created by the authors).

Many studies have demonstrated that bifidobacteria can improve CVD due to its high antioxidant activity, for example, *Bifidobacterium longum* subsp*. longum (B. longum)* CCFM752 prevented HP and aortic lesions in rats^26^, and its culture supernatant prevented Ang II-induced increases in O^2-^ and H_2_O_2_ in smooth muscle A7R5 cells of rat thoracic aorta^16^. Previously, it was observed that an increase in the number of bifidobacteria in the intestine was resulted in reduced cardiac risk ratios and atherosclerotic indices in broilers^27^. Numerous data on the mechanism of antioxidant action of bifidobacteria is available. First, iron-binding sites were identified in various bifidobacteria species, including *B. longum* NCC2705, which suggests its ability to chelate iron ions, thus inhibiting its catalytic effect on oxidation reactions (Fig. 1a)^23^. Catalase (CAT), which degrades hydrogen peroxide, has been widely shown to be a critical enzyme in the antioxidant defense system. Previous data showed that CAT activity was enhanced and NADPH oxidase (NOX) activity was inhibited in A7R5 cells preincubated with culture supernatant of *B. longum* CCFM752 (Fig. 1a)^16^. Interestingly, the enhancement of CAT activity was not due to changes at the transcriptional level but to the upregulation of related proteins at the translational level, as cellular transcriptome sequencing revealed that the 60S ribosomal protein L7a (Rpl7a) was upregulated and its expression was positively correlated with intracellular CAT activity. As for the suppression of NOX activity, it may be due to the decreased expression of Noxa1. Other studies have demonstrated that Nrf2 - Keap1 - ARE is a pivotal pathway in the antioxidant defense system (Fig. 1a). Upon exposure to oxidative stress, the active cysteine residues of Keap1 are modified, resulting in inactivation of Keap1. This allows Nrf2 to bind Keap1 to form a complex that is ubiquitinated. Next, the accumulated Nrf2 acts as a master regulatory transcription factor, activating phase II detoxification enzymes, drug transporters, and antioxidant enzymes, thereby exerting intracellular antioxidant defense^28^. Previous report have shown that bifidobacteria attenuate oxidative stress by promoting the dissociation of Nrf2 from Keap1 and facilitating the translocation of Nrf2 to the nucleus. (Fig. 1a)^29^. Multiple explanations have been proposed to account for the mechanism by which bifidobacteria induce the expression of Nrf2. The mechanism underlying the ability of *Bifidobacterium* to induce Nrf2 expression remained unclear until researchers directed their attention toward certain intestinal metabolites. The first is indole-3-lactic acid (ILA), a major catabolic metabolite of tryptophan produced by more than 50 *Bifidobacterium* species^30^, and in 2020 researchers found that ILA increased the mRNA expression of GPX2, SOD2, and NQO1, the target activator genes of Nrf2^31^. Second, an intestinal metabolite called sedanolide has recently been reported as a possible key effector in the activation of the Nrf2 pathway by bifidobacteria. Because the increase in sedanolide abundance in mice intestine, following administration of *B. longum* R0175, resulted in a significant upregulation of Nrf2 expression and Nrf2 pathway-related genes (NQO1, HO1)^32^. In addition, bifidobacteria metabolites, such as glutathione (GSH) and folic acid, also exert antioxidant benefits by maintaining the redox state of cells and increasing the antioxidant property of lipoproteins, respectively (Fig. 1a)^33^. It has also been proposed that the acid-producing effect of bifidobacteria could help maintain a low intestinal pH, limiting the proliferation of pathogenic bacteria, thus contributing to anti-oxidative stress (Fig. 1a)^33^. In summary, the anti-oxidative stress activity of bifidobacteria is realized by trapping metal ions, activating the antioxidant enzyme system and producing antioxidant metabolites which regulate intestinal microbiota.

### Immunoregulatory properties

Previous data have shown that inflammation is critical in the development of CVD, especially AS. Activation of various inflammatory signaling pathways and increased production of pro-inflammatory cytokines depict intensification of inflammatory response, which directly relates to CVD such as AS, I/R, and MI^34^. Furthermore, the inflammatory response could influence the prognosis of some CVD. For instance, a previous study demonstrated the association between the intensity of the inflammatory response and ventricular remodeling after MI^35^.

The immunoregulatory properties of bifidobacteria are well known and thus have long been used to treat several inflammatory conditions, such as allergies and necrotizing enterocolitis in premature infants. The accumulating knowledge about intestinal microbial functions has allowed researchers to focus on the relationship between bifidobacteria and CVD^36^. Liang et al.^14^ reported that *Bifidobacterium animalis* subsp*. lactis (B. lactis)* F1-7 enhanced the anti-inflammatory effect of krill oil, thereby alleviating inflammatory response in atherosclerotic mice more strongly compared to administration of krill oil alone. *B. lactis* has been reported in a randomized trial to significantly reduce the pro-inflammatory cytokines TNF-α and IL-6 in blood samples from patients with metabolic syndrome, which contributes to the reduction in cardiovascular risks in patients with metabolic syndrome^15^. Several specific molecular structures in bifidobacteria, such as secreted immunomodulatory proteins and peptides (Serpins, villi, peptidoglycan hydrolase TgaA) as well as extracellular polysaccharides (EPS), contribute to its anti-inflammatory properties (Fig. 1a)^37^. These specialized structures drive specific immunomodulatory processes by interacting with intestinal microbes or host cells. Specifically, production of serpin protects the bifidobacteria from adverse effects of host-derived proteases through anti-inflammatory activities^38^. Some villi (sorting enzyme-dependent hairs) could induce high levels of local inflammatory factors while inhibiting systemic inflammatory response, thus facilitating initial crosstalk between bifidobacteria and host cells^38^. Peptidoglycan hydrolase TgaA has been shown to induce IL-2 production in key cytokines in T cells^39^. EPS affects the differentiation of T cells into T helper cells thus regulating the levels of pro-inflammatory and anti-inflammatory cytokines. In addition, it was shown that bifidobacteria could trigger intestinal metabolite cross-feeding mechanisms and maintain intestinal homeostasis, which contributes to the maintenance of the intestinal immune system (Fig. 1a)^37,38^. Among the intestinal metabolites, butyrate is the potent anti-inflammatory agent that maintains the immune system and is involved in immune signaling by stimulating anti-inflammatory factors (TNF-β, IL-10) and inducing T-cell differentiation. Although bifidobacteria do not directly produce butyrate, the produced acetate can be used as a fermentation substrate for the indirect production of butyrate through cross-feeding interaction^40^. Strains of *Bifidobacterium* species also participate in tryptophan metabolism in the gut, while its metabolite indole-3-carbaldehyde (I3C) directly binds to AhR ligands involved in immune signaling and inhibits abnormal immune responses^41,42^. Although serotonin is well known as a central nervous system neurotransmitter, there is growing evidence that peripheral serotonin affects cardiovascular disease by influencing immune cell reactivity^43^. Previous studies have shown that bifidobacteria reduces serotonin levels by modulating key components of the serotonergic system in different host tissues, the number of intestinal chromophores and gene expression during serotonin synthesis and reuptake (THP1, SERT)^44–47^. In fact, these are far from allowing us to fully understand the role of bifidobacteria on the human immune system, as most of the current research is still at the cellular or animal level. Given the intricate nature of intestinal microorganisms, deciphering the immunomodulatory mechanisms of bifidobacteria within the gut presents a formidable task. It’s also important to acknowledge the potential influence, and even reversal, of bifidobacteria’s immunomodulatory effects due to the presence of other strains of bifidobacteria.

### Intestinal barrier

Intestinal barrier dysfunction is an undervalued risk factor for CVD^48^. The integrity of the intestinal barrier is maintained by the connections between intestinal epithelial cells, which consist of proteins such as tight junction (TJ) proteins, adhesion junction (AJ) proteins, gap junction proteins, and desmosomal proteins^49^. It is essential for intracellular communication and paracellular permeability that TJ proteins are located at the top of the intestinal epithelial cells (Fig. 1c). Once the TJ proteins are impaired, bacteria and bacterial products such as lipopolysaccharide (LPS) in the intestines leak and enter the systemic circulation (Fig. 1a)^48^. There are accumulating reports on the association between the translocation of LPS and CVD. Abnormal elevation of serum LPS has been observed in AS, HP, chronic heart failure (CHF), and MI^48^. Indeed, impaired intestinal barrier, endothelial dysfunction, and pro-inflammatory response induced by LPS translocation have been shown to seriously affect the development of CVD.

It should be emphasized that the abundance of bifidobacteria directly impacts the integrity of the intestinal barrier. A previous study using mouse models demonstrated that a high-fat diet (HFD) damages the intestinal barrier by reducing the number of bifidobacterial^50^. The effects of bifidobacteria on TJ and AJ proteins have been demonstrated in recent studies. One study acknowledged that administration of bifidobacteria inhibited LPS translocation by increasing serum glucagon-like peptide-2 (GLP-2) to upregulate TJ proteins (occludin ZO1 and occludin) (Fig. 1a)^17^. As metabolites of bifidobacteria, short-chain fatty acids (SCFAs) are reported to protect intestinal barrier function by promoting assembly of TJ proteins and inhibiting activation of inflammatory vesicles (Fig. 1c). These data demonstrate that bifidobacteria is indeed beneficial in maintaining the intestinal barrier; and thus has the potential to be used in the repair of “leaky gut”. However, the precise mechanism underlying the protective impact of bifidobacteria on the intestinal barrier remains incompletely understood. This includes its interplay with structures like the villi and crypts of the intestinal epithelium, as well as whether changes in junction proteins are linked to the attenuation of inflammatory processes.

### Gut microbiota metabolites

Intestinal microbial metabolites are novel risk factors for cardiovascular events^51^. A gastrointestinal tract is integral in digestion and absorption, and gut microbiota creates a bridge between diet and host. The gut microbiota produces an abundance of small-molecule metabolites when involved in the co-metabolism of food or exogenous substances, some of which are critical in the transmission of information between the host gut and distant organs, such as trimethylamine N-oxide (TMAO), SCFAs, conjugated fatty acids and secondary bile acids^52,53^. Recently, there has been a lot of interest on the causal relationship between the small molecules and CVD, and it has been found that these small-molecule metabolites play a role in the development and progression of CVD^54^.

#### Trimethylamine N-oxide (TMAO)

TMAO is a potential risk factor for chronic diseases, especially CVDs. The intake of red meat, milk, poultry, and eggs increases the body’s choline or trimethylamine products, which are metabolized by the intestinal microbiota to produce trimethylamine (TMA). TMA undergoes intestinal absorption and portal vein transport and is then oxidized to TMAO by flavin-containing monooxygenases (FMO) 1 and 3 in the liver (Fig. 1c)^55^. TMAO has been shown to be a potential promoter of CVD, especially AS^56^. Several mechanisms of TMAO-promoting AS have been proposed, which include promotion of the expression of inflammatory factors, breakdown of the balance of cholesterol metabolism, and induction of thrombosis^57^. It is worth mentioning that TMAO has been proven to predict the risk of CVD in multiple clinical cohorts, and has demonstrated uninterrupted prognostic value in a variety of adverse cardiac events (including coronary artery disease, HF, MI, death)^58–62^.

In addition, it has been hypothesized that bifidobacteria could down-regulate TMAO levels in vivo, which has been confirmed by findings from multiple studies. In the previous study on the mechanism of resveratrol against AS, it was demonstrated that the level of bifidobacteria in the intestinal microbiota of mice increased, accompanied by a decrease in the TMAO level^63^. This negative correlation between bifidobacteria and TMAO levels led to the exploration of more profound interactions. The TMAO-lowering properties of bifidobacteria have been explained by several mechanisms (Fig. 1c). A study conducted in choline mice showed that three bifidobacteria, including *Bifidobacterium breve* (*B. breve*) Bb4*, B. longum* BL1, *and B. longum* BL7, significantly decreased plasma TMAO levels and restored the abundance of some gut microbial species, while maintaining the activity of FMO^18^. This finding suggests that bifidobacteria may be reducing TMAO levels by modulating gut microbial abundance. Similarly, another study reported that administration of *B. lactis* F1-3-2 decreased plasma TMAO, which was not dependent on the regulation of FMO. Whether, it may have directly degraded TMA or adjusted the intestinal microbiota structure remains to be determined^64^. In addition, bifidobacteria may be antagonizing some strains responsible for synthesizing TMAO precursor molecules, such as *Clostridium difficile*^65^. Some probiotics have been proposed to reduce TMAO levels by altering miRNAs and modulating metabolomic profiles^66^, but data on bifidobacteria are still scarce and more targeted experimental evidence is needed.

#### Short-chain fatty acids (SCFAs)

Previously, data on the metabolism of SCFAs in CVD remained limited. However, with the growing evidence, it was shown that SCFAs are important in regulating cardiovascular functions^67^. In HP, both an increase in propionic and acetic acids induces vasodilation, and butyrate has been shown to relieve HP by inhibiting the angiotensin system in the kidney^68^. The properties of SCFAs such as immunomodulation, antioxidant stress, and improvement of lipid metabolism, are of great significance to the treatment of AS^69,70^. In addition, SCFAs act on the nervous system to protect the heart from injury and maintain its functions. A previous study showed that butyrate reversed autonomic imbalance in I/R rats and improved cardiovascular function by targeting the paraventricular nucleus and superior cervical ganglia^71^. It has also been established that butyrate may prevent ventricular arrhythmias after MI by inhibiting sympathetic remodeling^72^. SCFAs are a particular metabolite whose impact on the development of CVD is of far-reaching significance.

Human dietary fiber is degraded into organic acids by intestinal microflora, gas, and a large number of SCFAs^73^. Acetic acid, propionic acid, and butyric acid account for 90% of the SCFAs^74^. It is important to note that acetic acid is the main end-product of the metabolism of bifidobacteria. Bifidobacteria could indirectly increase butyric acid levels in the gut by cross-feeding interactions which enhance intestinal colonization of other butyric acid-producing commensal microorganisms (Fig. 1c), such as *Faecalibacterium prausnitzii*^75^ and *Eubacterium hallii*^76^. However, cross-feeding interactions between bifidobacteria and butyrate-producing bacteria vary from species to species, ranging from symbiotic to competitive relationships for energy substrates, which are largely related to the ability of bifidobacteria to degrade energy sources^77^. Currently, studies on the ability of different *Bifidobacterium* strains to degrade energy sources need to be deepened and continuously improved.

## Indirect improvement of cardiovascular disease

### Obesity

Obesity is associated with numerous severe health consequences, with cardiovascular disease (CVD) being a primary concern. It contributes to the development of CVD and increases CVD mortality^78^. Recent data suggest that severe obesity increases the risk of cardiovascular-related incidents in varying degrees: it increased the risk of HF by about 4-fold and increased the risk of coronary heart disease and stroke by nearly 2-fold^79^.

#### Appetite

Recent studies have suggested that bifidobacteria are involved in energy homeostasis and appetite regulation in the central nervous system (CNS) by improving levels of hormones such as leptin and gastrin^80,81^.

**Fig. 2:**
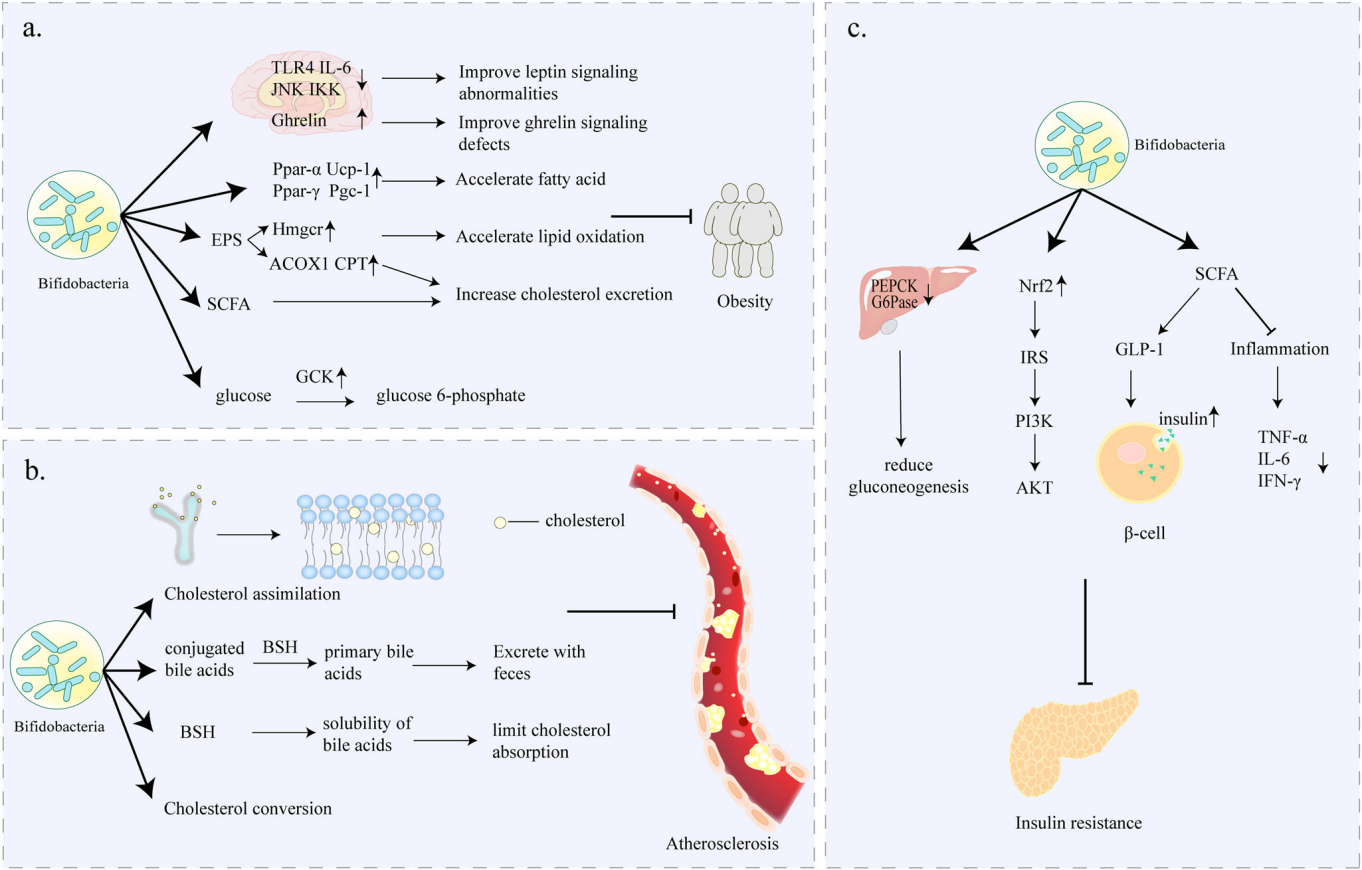
Indirect Improvement of CVD. **a** *Bifidobacterium* can control appetite by affecting the transmission of leptin and gastric hunger signaling pathways; it can also reduce obesity by inhibiting the disordered lipid metabolism process and reducing the impaired glucose metabolism in obese hosts in multiple ways. **b** *Bifidobacterium* can reduce the development of hyperlipidemia through cholesterol assimilation; high BSH ability to increase cholesterol excretion in the feces and limit cholesterol absorption, thus promote cholesterol conversion. **c** *Bifidobacterium* can reduce glucose metabolism by targeting hepatic gluconeogenesis genes, restoring insulin signaling pathway; producing SCFA to secrete GLP-1 and regulating islet beta cell growth and modulating the target inflammation to improve insulin resistance and reduce diabetes. (The figure does not contain any third-party material, the figure and each of the elements in the figure were created by the authors).

Leptin, a peptide hormone, is associated with the CNS’s perception of energy balance and food intake^82^. Leptin works by reducing dietary intake and body weight, so as the body fat increases, leptin continues to rise^83^. In chronically obese people, persistently had high levels of leptin in the blood reduce the sensitivity of leptin receptors in the hypothalamus, a phenomenon widely known as leptin resistance. Leptin resistance is characterized by a strong appetite, reduced energy expenditure and obesity. The mechanisms of leptin resistance include stimulation of inflammatory factors leading to abnormal signaling, reduced efficiency of blood-brain barrier transport, and receptor mutations. A study by Renata et al.^81^ demonstrated that after giving gastric gavage of probiotics containing *B. bifidum* to HFD-fed mice, the leptin resistance of obese mice was significantly improved, which was reflected in the significantly reduced food intake in mice. This may be due to the fact that probiotics containing *B. bifidum* significantly reduce mRNA levels of pro-inflammatory molecules TLR4 and IL-6 and expression of JNK and IKK proteins in the hypothalamus, thereby improving leptin signaling abnormalities (Fig. 2a). The molecular interaction between bifidobacteria and leptin remains unclarified. However, it has been demonstrated that SCFAs stimulate the expression of leptin in adipocytes by activating the free fatty acid receptor 3 (FFAR3)^84^, which provides a basis for future studies on the association between bifidobacteria and leptin.

Besides reducing appetite by improving impaired leptin signaling pathways, bifidobacteria also improve ghrelin signaling pathways. Gastrin is an endocrine hormone produced in the gastric mucosa, which is responsible for the regulation of appetite and energy expenditure^85,86^. Specifically, ghrelin is secreted in large quantities during starvation, which then enters the blood circulation and reaches the center. Thereafter, it acts on the hypothalamic and midbrain limbic circuits to control dietary behavior and food intake^87^. For instance, a previous study showed that when non-obese individuals ate for 30 minutes, ghrelin was immediately suppressed by 39.5 % and continued to decline until it returned to baseline^88^. In contrast, for obese individuals, food intake did not seem to cause the ideal decrease in ghrelin^88^. Such postprandial ghrelin passivation is usually one of the reasons for excessive food intake in obese people. Previous studies using animal models showed that serum ghrelin levels were negatively correlated with the abundance of some bacterial populations in the gut microbiome, including the *Bifidobacterium* species^89^. Some scholars have explored the effect of bifidobacteria on human anti-obesity development. Findings from the studies showed that *B. longum* APC1472 significantly increased the ghrelin activity in obese individuals, which may demonstrate that *B. longum* APC1472 improves ghrelin signaling defects (Fig. 2a)^80^.

#### Lipid and glucose metabolism

Apart from the appetite in obese people, metabolic abnormalities directly enhance the process of obesity. It has been shown that obesity-related metabolic disorders are often accompanied by a decrease in the genus *Bifidobacterium* in the intestines^90^. Nevertheless, accumulating experimental evidence suggests that bifidobacteria supplementation is beneficial in inhibiting lipid metabolism process in obese host disorders in multiple ways.

In general, in non-obese people, there is maintenance of a dynamic balance between fat consumption and production. However, due to long-term unhealthy lifestyles or some disease factors, there is destruction of the lipid homeostasis in the body, and the decomposition of fat is far less than the production of fat, resulting in excessive accumulation of lipids and eventual obesity^91^. A variety of bifidobacteria, such as *Bifidobacterium adolescentis (B. adolescentis)* and *Bifidobacterium animalis* subsp*. animalis (B. animalis)*, showed a tendency to restore lipid profiles (including LDL-C, HDL-C, TC, triglyceride (TG)) to normal levels^19,92^. It has been demonstrated that bifidobacteria regulate fat accumulation by upregulating mRNA expression of thermogenic genes and lipolytic enzymes, and inhibiting activation of lipogenic genes. For example, *B. adolescentis* was observed to upregulate mRNA expression of Ucp-1, Pgc1-α, Ppar-γ, and Ppar-α genes in HDF-fed mice, to produce fatty acids which promote lipid metabolism in brown adipose tissue (Fig. 2a)^19^. Ppar-α upregulates the expression of enzymes responsible for mitochondrial fatty acid oxidation, while Ppar-γ activation triggers brown adipocyte differentiation and adipogenesis^93^. These results suggest that acceleration of fatty acid conversion is one of the ways bifidobacteria use to improve fat metabolism (Fig. 2). In addition, some bifidobacteria may also improve lipid metabolism by producing EPS, a substance with potential health benefits^94^. Another study demonstrated that the expression of acyl-CoA oxidase 1 (ACOX 1), carnitine palmitoyl transferase 1A (CPT 1A), and 3-hydroxy-3-methyl-3-glutaryl-CoA reductase (Hmgcr) of Diet-Induced Obese Mice was significantly upregulated by the application of *B. animalis* IPLA R1, which produce EPS (Fig. 2a)^92^. ACOX 1 and CPT 1A are rate-limiting enzymes in the fatty acid oxidation pathway, while Hmgcr is a rate-limiting enzyme in cholesterol synthesis^95^. These data suggest that *B. animalis* IPLA R1 can inhibit fat accumulation in the liver by accelerating lipid oxidation and increasing cholesterol excretion (Fig. 2a). Similarly, SCFAs, as intestinal microbial metabolites, can be directly involved in improving lipid metabolism because of their ability to increase lipid oxidation (Fig. 2a)^69,96^.

Numerous studies have indicated that the regulation of lipid metabolism by bifidobacteria is often accompanied by the normalization of blood glucose levels^97–99^. Parallel to lipid metabolism disorders, glucose metabolism disorders are also closely related to obesity. A study by Cano PG et al.^99^ found that seven weeks of continuous administration of *Bifidobacterium pseudocatenulatum (B. pseudocatenulatum)* CECT 7765 reduced blood glucose levels in obese HFD-fed mice by 17%, demonstrating its ability to increase glucose tolerance. The improvement of glucose metabolism parameters by bifidobacteria may be associated with the activation of glucokinase (GCK) (Fig. 2a)^100^. It is worth noting that GCK has better control over the rate of insulin secretion in pancreatic β-cells, and thus small changes in its activity can cause fluctuations in the insulin secretion threshold and affect glucose homeostasis^100^. Previous systematic analyses showed that yogurt fermented *B. longum* 070103 successfully activates GCK and thus significantly reduces fasting blood glucose and improves glucose tolerance as well as insulin resistance^101^. Moreover, specific regulation of the abundance of crucial intestinal microbes, reduction of the levels of metabolites such as 3-indolyl sulfate and 4-hydroxybutyric acid to alleviate glucose metabolic disorders could be included in the mechanism of bifidobacteria hypoglycemic activities^101^.

### Hyperlipidaemia (HLP)

HLP is defined as excessive blood lipids (the sum of all lipids in the plasma) due to abnormal fat metabolism or transport. Abnormal levels of one or more lipids in the plasma often lead to HLP, and include increased total cholesterol (TC), TG, low-density lipoprotein cholesterol (LDL-C), and decreased high-density lipoprotein cholesterol (HDL-C)^102^. This explains why HLP presents as hypercholesterolemia, hypertriglyceridemia, or both (mixed HLP). However, lack of intervention eventually leads to CVD, regardless of the kind of HLP. Early intervention of dyslipidemia is essential for primary and secondary prevention of CVD, especially AS and coronary heart disease.

#### Hypercholester

Cholesterol is one of the subtypes of lipids which is transported to the whole body in form of lipoproteins. Lipoproteins can be divided into LDL-C, very low-density lipoprotein cholesterol (VLDL-C), and HDL-C, which perform unique functions in the body. LDL-C carries cholesterol into the artery and binds to macrophages on the arterial wall. Macrophages absorb cholesterol and gradually develop into foam cells, burying the hidden danger of AS. LDL-C is, therefore, generally considered “bad” cholesterol. On the contrary, HDL-C transports cholesterol accumulated in the arteries to the liver for metabolism, thereby preventing CVD. It has been suggested that every 10 mmol/L reduction in LDL cholesterol reduces the risk of CVD and mortality by 22%^102^. Adverse reactions or contraindications to statins and fibrates currently used in clinical practice are plaguing doctors and patients^103^. The role of bifidobacteria in lowering cholesterol is constantly emphasized, not only because of its effectiveness but also its diverse mechanisms of action in lowering the cholesterol. Cholesterol assimilation occurs when *Bifidobacterium* binds cholesterol to the surface of the cell and absorbs it into the membrane’s phospholipid bilayer (Fig. 2b)^104^. Different *Bifidobacterium* strains showed varying degree of assimilation abilities. For example, under the same experimental conditions, the adsorption rate of *Bifidobacterium bifidum (B. bifidum)* MB109 to cholesterol was 52%, while that of *Bifidobacterium longum* subsp*. infantis (B. infantis)* ATCC 15697 was 34%^105^. Secondly, bifidobacteria’s bile salt hydrolase (BSH) activity enables it to reduce cholesterol by participating in the metabolism of the bile acids. More specifically, *Bifidobacterium* hydrolyzes conjugated bile acids into primary bile acids through its high BSH capacity, making them more easily excreted in feces (Fig. 2b). The reduction of bile salts and the loss of bile acids stimulate the liver to increase cholesterol conversion, which decreases serum cholesterol levels. These findings were confirmed by Al-Sheraji and Jiang in an in vivo study using rats^20,106^. BSH activity has also been shown to limit cholesterol absorption by reducing the solubility of bile acids (Fig. 2b)^107^. Thirdly, cholesterol conversion also contributes to the removal of cholesterol by bifidobacteria (Fig. 2b). Another study observed an increase in coprostanol in cholesterol-rich cells treated with *B. bifidum* PRL2010, accompanied by upregulation of BBPR 0519, which is predicted to encode the aldehyde/ketone reductase which catalyzes the conversion of cholesterol to coprostanol^108^.

#### High triglycerides (TG)

More than 30 years ago, the negative effect of high TG to CVD was comparable with that of high TC^109^. Although the advent of statins has led to more focus on lowering LDL-C over time, there has been a renewed interest in TG^110^. It has been proved that the size of a lipoprotein can enter the arterial wall when TG concentration is slightly or moderately elevated (2–10 mmol/L), thus increasing the risk of CVD^111,112^. In contrast, when its concentration is seriously elevated (>50 mmol/L), the lipoproteins become too large to enter the arterial wall, which affect people’s judgment of the TG^113^. Although there may be more than one mechanism for TG in CVD, there is one mechanism that has been discussed most clearly: high TG leads to increased concentrations of residual cholesterol, which enters the arteries, promotes inflammatory episodes and foam cell formation, which ultimately increases the risk of CVD and death^114^.

Bifidobacteria were negatively correlated with TG levels. Further clinical studies have shown that supplementation with bifidobacteria or intake of functional foods containing bifidobacteria could reduce serum TG levels^115,116^. An improvement in the overall lipid profile usually accompanies TG-lowering effect of bifidobacteria. The TG-lowering mechanism of the bifidobacteria is less precise than that of cholesterol-lowering. However, the benefits of bifidobacteria and good tolerance in reducing TG make it worthy of consideration in the therapy of lipid abnormalities.

### Type 2 diabetes mellitus (T2DM)

Diabetes is an established risk factor for CVD such as HF, coronary heart disease, stroke, and atrial fibrillation^117^. It is estimated that 2–4 times more diabetic patients die from CVD than non-diabetic patients, and at least 68% of diabetics over 65 years old die from various forms of CVD^118^. More than two-thirds of patients with T2DM are reported to have HP^119^. According to a meta-analysis report, the risk of HF was 1.11 (95% CI, 1.04–1.17) for every 1 mmol/L (≈18 mg/dL) increase in fasting blood glucose, indicating a positive correlation between fasting blood glucose and HF^120^.

Recently, the unique benefits of bifidobacteria in the treatment of T2DM have been validated in both animal studies and clinical trials. More studies have revealed the mechanisms underlying this beneficial role, and several phased results have been achieved. Studies have claimed that bifidobacteria have a significant effect on improving insulin resistance (IR) in patients with T2DM, and some strains are even more effective than metformin^21,29,121,122^. The experimental studies by Zhang et al.^29^ showed that bifidobacteria utilize several pathways to improve insulin sensitivity. First, bifidobacteria may alleviate IR by targeting hepatic gluconeogenesis genes to reduce gluconeogenesis (Fig. 2c). The study used *B. animalis* 01 in T2DM rats, and showed that *B. animalis* 01 down-regulated phosphoenolpyruvate carboxykinase (PEPCK), glucose-6-phosphatase (G6Pase), and upregulated the expression of Nrf2, IRS-2, PI3K, and AKT-2 genes (Fig. 2c)^29^. Researchers have suggested that *B. longum* BL12 and *B. lactis* HY8101 down-regulate PEPCK and G6Pase in the liver of T2DM mice^21,121^. Nrf2 is a protective factor against oxidative damages. Activation of the Nrf2 inhibits IRS-2 phosphorylation and thus increases the expression of downstream signals such as PI3K and AKT (Fig. 2c). The IRS - PI3K - AKT pathway is critical in hepatic insulin signaling. Therefore, enhancement of the antioxidant capacity is another effective way adopted by bifidobacteria to restore insulin signaling and repair IR.

Inflammation is known to be associated with induction of IR and development of T2DM^123^. Due to its anti-inflammatory properties, *B. adolescentis* strains have recently been found to alleviate IR. The diabetic state of T2DM mice was improved after administration of *B. adolescentis*. The pro-inflammatory factors including TNF-α, IL-6, and IFN-γ were significantly inhibited, and the concentrations of butyric and propionic acids were significantly increased (Fig. 2c)^122^. Based on the high association between the elevated concentrations of SCFAs caused by these strains and their effects on blood glucose concentrations, Qian^122^ hypothesized that the ameliorative diabetic effects of adolescent bifidobacteria are mediated through the bifidobacteria-gut microbiota-SCFAs-inflammation axis. *B. lactis* GCL2505, which has been evaluated for improving diabetes, appears to regulate SCFAs (particularly acetate) levels^124^. In addition, elevated SCFAs stimulate GPR43, leading to the secretion of GLP-1, which regulates β-cell growth, stimulates glucose-dependent insulin release, and inhibits glucagon secretion (Fig. 2c)^124^.

## Current applications in cardiovascular disease

Clinical studies on applications of *Bifidobacterium* species in CVD are summarized in Table 1. The studies vary by subject, sample size, bacteria, product, dosage, and study design.

**Table 1:** The cases of bifidobacteria in CVD.

Subject and sample size	Bacteria	Products	Dasage & duration	Results
HP women^40,127^	*Lactobacillus casei LPC-37, Lactobacillus rhamnosus HN001, Lactobacillus acidophilus NCFM* and *B. lactis HN019*	Probiotic cocktail Probiatop^®^	1 × 10^9^ CFU/day, 8 weeks	Significantly reduced fasting blood glucose and cholesterol levels and increased HDL-cholesterol; Decreased systolic blood pressure levels by about 5 mmHg and diastolic blood pressure decreased by about 2 mmHg. The reduction in levels were insignificant
Grade 1 HP patients	*Lactobacilli paracasei, plantarum, acidophilus,* and *delbrueckii; B. longum, infantis* and *breve; Streptococcus thermophilus*	Vivomixx^®^	9 × 10^11^ CFU/day, 8 weeks	Average reduction in systolic blood pressure levels was 3.8 mmHg
Healthy male adults^32,117^	*B. longum* BL1	Fermented yogurt	>3 × 10^8^ CFU/day, 8 weeks	Significant reduction in serum total TC levels
Patients with mild hypercholesterolemia (180–220 mg/dL)^40,136^	*Lactobacillus acidophilus* La5*, Lactobacillus casei* TMC*, B. lactis* Bb12	Probiotic milk formula	6 × 10^7^ CFU/day, 10 weeks	TC (8.1%) and LDL-c (10.4%) levels decreased
Healthy young adults^30,137^	*B. lactis* BB-12	Yogurt smoothie	3.16 × 10^9^ CFU/day, 4 weeks	No improvement in lipid and lipoprotein levels
Patients with T2DM and low-density lipoprotein cholesterol (LDL-C) > 2.6 mmol/L^59,138^	*Lactobacillus acidophilus* La5 and *B. lactis* Bb12	Fermented yogurt	>3.57 × 10^8^ CFU/day, 6 weeks	TC decreased by 4.54% while LDL-C decreased by 7.45%. The decrease in TC and HDL-C levels were insignificant
Healthy young males^40,140^	*Lactobacillus acidophilus, Lactobacillus rhamnosus* GG*, B. animalis* and *B. longum*	Powder	*B. animallis*, Bi-04 1 × 10^9^ CFU/day, *B. longum* 2.5 × 10^9^ CFU/day, 4 weeks	A higher proportion of participants in the probiotic group showed a decrease in TMAO after intervention
Healthy adults^27,141^	*B. lactis* LKM512	Powder sticks	6 × 10^9^ CFU/day, 12 weeks	The TMA-producing bacteria, fecal TMA concentration, and BMI in the probiotic group were lower than those in the placebo group; serum TNF-α concentrations only decreased in the probiotic group
Patients with overweight, diabetes, and coronary heart disease patients^59,147^	*Lactobacillus acidophilus* strain T16*, Lactobacillus casei* strain T2, and *B. bifidum* strain T1	Capsule	2 × 10^9^ CFU/day, 12 weeks	Significantly reduced serum high-sensitivity C-reactive protein (hs-CRP), plasma malondialdehyde (MDA), and significantly increased nitric oxide (NO) levels
Patients with CHF^89,169^	*Lactobacillus acidophilus* La5 and *B. lactis* Bb12	Fermented yogurt	3 × 10^9^ CFU/day, 10 weeks	Significantly increased sTWEAK levels
Patients with CHF^89,170^	*Lactobacillus acidophilus* La5 and *B. lactis* Bb12	Fermented yogurt	3 × 10^9^ CFU/day, 10 weeks	Significant reduction of serum oxLDL levels
Gastric cancer patients with coronary heart disease and HF complications^16,171^	*B. longum, Lactobacillus acidophilus* and *Enterococcus faecalis*	Capsule	≥4.0 × 10^7^ CFU/day, 4 weeks	Endotoxin, D-lactic acid, procalcitonin decreased, intestinal mucosa improved significantly

### Hypertension (HP)

HP is a chronic disease that is characterized by continuously high arterial blood pressure levels. Long-term HP is the most significant risk factor for coronary artery disease, stroke, HF, atrial fibrillation, and other CVDs. A 20 mmHg increase in systolic blood pressure and a 10 mmHg increase in diastolic blood pressure are associated with a two-fold increase in the risk of death from stroke, heart disease, or other vascular diseases^125^. Based on evidence from CVD attribution analyses, a rightward shift in blood pressure distribution resulting in major cardiovascular diseases in humans has been proposed^126^.

A link between reduced bifidobacteria abundance and increased blood pressure incidences in children with type 1 diabetes mellitus (T1DM) has been proposed^13^. In this study, children were assigned into three groups: healthy control group (HC, *n* = 5), T1DM group with normal blood pressure levels (T1DM-Normo, *n* = 17), and type 1 diabetes group with elevated blood pressure levels (T1DM-HBP, *n* = 7)^13^. Analysis of gut microbiota for each group revealed that bifidobacteria abundance in the guts of children in the T1DM-HBP group were significantly lower than in the other two groups^13^. Another trial indicated that supplementation with probiotics containing *B. lactis* HN019 reduced systolic blood pressure levels by 5 mmHg and diastolic blood pressure levels by 2 mmHg in women with arterial HP, with effective improvements in lipid metabolism as well as fasting glucose levels^127^.

In Germany, 100 grade 1 HP patients were invited to participate in probiotic intervention trials^128^. After 8 weeks of dynamic nocturnal blood pressure monitoring and evaluation of fecal microbiome composition as well as immune cell phenotypes, it was proposed that the mechanisms by which bifidobacteria reduce HP involve improving immune cell homeostasis by transforming dietary metabolic components^128^. This hypothesis has been tested in rat models. In deoxycorticosterone acetate (DOCA) salt hypertensive rat models, *B. breve* CECT7263 alleviated HP by increasing acetate concentrations in the gut and reducing TMA, restoring Th17/Treg immune homeostasis and suppressing vascular NADPH oxidase activities^129^. In spontaneously hypertensive rat (SHR) models, *B. breve* CECT7263 suppressed the elevated blood pressure levels by increasing the number of butyrate-producing bacteria, preventing Th17/Treg dysregulation, reducing endotoxemia, and improving endothelial dysfunctions^130^.

### Atherosclerosis (AS)

Clinically, AS is an intimal disease in which fatty deposits form plaques in inner layers of arteries^131^. Then, growth of plaques leads to thrombosis and bulging in arteries. Fibrous tissue proliferation and calcium deposition accelerates arterial wall thickening and hardening^131^. Finally, the narrowing or blockage of arterial lumen leads to ischemia and necrosis of tissues and organs supplied by the artery^131^. Physiologically, the processes involved in AS development are complex and slow, involving many pathological changes, including hypercholesterolemia, inflammation, oxidative stress, and TMAO^66,131,132^. Even though the cholesterol-lowering, anti-inflammatory, anti-oxidative stress, and TMAO-modulating effects of bifidobacteria have been previously reported, the corresponding mechanisms are described in detail in Milad’s review^133^. However, more preclinical and clinical trials should be performed to improve AS through bifidobacteria supplementation.

Randomized trials involving individuals with mild to moderate hypercholesterolemia revealed significant cholesterol-lowering effects of bifidobacterial^134^. Thirty-two adult males were randomized into two groups and administered with 3 × 100 ml/day of ordinary yogurt or fermented yogurt for 4 weeks^116^. A decrease in TC levels was established in the *B. longum* BL1 fermented yogurt group and the effects were more pronounced in individuals with moderate hypercholesterolemia (TC > 240 mg/dl)^116^. Another randomized trial revealed that in mild hypercholesterolemia patients with TC levels between 180 and 220 mg/dl, 10 weeks of a formula containing *B. lactis* Bb12 reduced their TC and LDL-C levels by 8.1% and 10.4%, respectively^135^. A clinical trial involving healthy young people showed that bifidobacteria improved lipoprotein profiles in hypercholesterolemia patients but not normal cholesterol levels^136^. In addition, some valuable clinical data showed that bifidobacteria improves TC levels T2DM patients^137,138^.

An animal study investigating the effects of probiotics on plasma TMAO revealed that 7 of 16 *Bifidobacterium* strains significantly reduced plasma TMAO concentrations, and that the plasma TMAO reduction rate for *B. longum* BL1 was as high as 30.89%^18^. Current clinical trials have shown that bifidobacteria inhibits TMAO levels. In a previous study, TMAO levels were found to be elevated in 40 healthy young men (20–25 years) that had been subjected to a phosphatidylcholine challenge test^139^. Then, they were randomized into two groups, one of which received probiotic intervention^139^. The TMAO levels for most participants in the probiotic group decreased, however, the decrease was insignificant^139^. A similar randomized double-blind trial involving 27 healthy volunteers (mean age 47.1 years) obtained contrasting results^140^. In this study, participants were assigned to receive either *B. lactis* LKM512 or placebo^140^. After 12 weeks, fecal TMA concentrations and the abundance of TMA-producing bacteria were significantly low in the bifidobacteria group, relative to the placebo group (*p* < 0.05)^140^. It is important to note that since there is a positive correlation between plasma TMAO levels and age, age of the subjects may have been a critical factor leading to different outcomes of the two experiments^141,142^. This may be due to the fact that expressions of FMO3 isoforms increased with age in clinical models, which promoted TMA transformation to TMAO^143^. Overall, studies should elucidate on the link between bifidobacteria and TMAO.

The potential mechanisms by which bifidobacteria affect atherosclerotic CVD via low-grade inflammation have been discussed^144^. In the past 5 years, clinical trials have reported the positive effects of bifidobacteria on inflammation and oxidative stress in diabetic patients with coronary heart disease^145,146^. One of the trials involved 60 overweight, diabetic, and coronary heart disease patients aged 50–58 years and it aimed at assessing the effects of synbiotics (including bifidobacteria) on inflammatory biomarkers of carotid intima-media thickness and oxidative stress^146^. After 12 weeks of treatment, bifidobacteria significantly reduced hsCRP as well as plasma malondialdehyde levels, and significantly increased nitric oxide (NO) levels^146^. In Poland, probiotic supplements containing various *Bifidobacterium* strains were shown to reduce IL-6, TNF-α, and thrombomodulin levels in postmenopausal obese women and effectively improved arterial stiffness^147^.

### Myocardial infarction (MI)

Clinically, MI, known as myocardial ischemic necrosis, involves a rapid reduction or interruption of coronary blood supply due to coronary arterial disease (such as AS, spasm), leading to acute and persistent myocardial ischemia in the myocardium of coronary supply sites, and ultimately resulting in myocardial necrosis.

Previously, studies have reported a low gut abundance of bifidobacteria in rats^148^ and humans^149^ with MI. However, they did not conclusively determine whether bifidobacteria are cardioprotective against MI. The direct association between bifidobacteria and MI was first reported by Lam et al. in 2012. Lam et al.^150^ reported that myocardial infarct sizes were reduced by 29% in MI rats that had been fed on Goodbelly for 14 days. Goodbelly is a commercially available probiotic juice containing two probiotics, *Lactobacillus plantarum* 299v and *B. lactis* Bi-07. Due to the absence of relevant evidence, it has been postulated that protective effects of this probiotic are as a result of reduction in serum leptin levels. Danilo et al.^151^ found that administration of *B. lactis* B420 for 4 weeks or 7 days significantly mitigated myocardial infarct sizes in mice following I/R. The epigenetic mechanisms of *B. lactis* B420 against MI were also identified. Further, Danilo et al. explained that the anti-MI effects of *B. lactis* B420 were achieved by suppressing the levels of inflammatory factors and accelerating transitions to M2-type macrophages via the mediatory effects of anti-inflammatory T-regulatory immune cells. Their findings are in tandem with those of Jafar Sadeghzadeh et al. who found that oral administration of a probiotic combination formulation containing *B. breve* exerted cardioprotective effects on rats with infarct-like myocardial injuries by attenuating TNF-α and inhibiting oxidative stress^152^.

Depression-like behaviors and development of depression are frequent in MI patients^153^. Bifidobacteria have been shown to reduce the development of depression-like behaviors after MI in animal models^154–156^. Since increased apoptosis was observed in various limbic system structures after MI to varying degrees, Girard et al.^157^ first proposed that prophylactic supplementation with *B. longum* and *Lactobacillus* reduces apoptosis. They confirmed this hypothesis in rat models and postulated that this combination of probiotics exerts anti-inflammatory effects leading to inhibition of apoptosis in the limbic system^157^. In addition to preventive effects, probiotic supplementation after ischemia-reperfusion has been shown to maintain its beneficial effects while improving depression-like behaviors after MI^155^. Later, scholars determined that *B. longum* plays more beneficial roles in probiotic combinations to improve depression after MI^156^. A randomized, double-blind, placebo-controlled clinical trial revealed the beneficial effects of probiotic supplementation (*Lactobacillus rhamnosus*) on depressive symptoms, inflammation, and oxidative stress in MI patients^158^. Bifidobacteria, which has shown excellent anti-depressive effects after MI in preclinical studies, may also hold up in clinical trials, although the link has yet to be established.

### Heart failure (HF)

Since HF is not an independent disease, its definition in academia is inconclusive. The American Heart Association defines it as a complex clinical syndrome resulting from various cardiac structural or functional diseases that impair ventricular filling or ejection capacities^159^. Long-term HP increases cardiac load, leading to myocardial hypertrophy, remodeling, and HF^160^. Coronary atherosclerotic heart disease (coronary heart disease) results in long-term heart ischemia and hypoxia, leading to gradual weakening of heart contractions, thereby inducing HF^161^. Physiologically, MI is a risk factor for HF in coronary heart disease. This is because MI leads to a sharp decrease in myocardial contractility and to a significant decrease in cardiac pumping volume. Thus, after infarction, the myocardium becomes fragile, aggravating HF development^162^. HF is usually the common end for multiple CVDs^163^. The 5-year survival rate for HF patients is only 45%, indicating that new prevention and treatment strategies are urgently required to improve on its prognosis^164^.

Soluble tumor necrosis factor-like weak inducer of apoptosis (sTWEAK) is an independent predictor of mortality risk in CHF, and non-ischemic HF, implying that it is a potential new cardiovascular biomarker^165–167^. Compared with healthy individuals, the concentrations of sTWEAK in patients with congestive HF, coronary artery disease, and AS have been shown to be suppressed^167^. A triple-blind clinical trial randomly assigned 90 CHF patients into two groups receiving probiotic yogurt containing bifidobacteria or ordinary yogurt for 10 weeks^168^. Compared with ordinary yogurt, probiotic yogurt increased serum sTWEAK levels in CHF patients^168^. The increase in serum sTWEAK levels may have been because probiotic intake reduces inflammation, thereby downregulating Fn14, the only receptor for sTWEAK, which prevents sTWEAK from binding Fn14. In addition, a randomized triple-blind clinical trial showed that probiotic yogurt significantly reduced serum ox-LDL levels, which might be related to the increase in total antioxidant capacities through multiple pathways (production of antioxidant metabolites, modification of MAPK, NF-κB, and other pathways, as well as regulation of ROS-producing enzymes)^169^.

In addition to patients with HF alone, a previous clinical trial focused on gastric cancer patients with coronary heart disease and HF complications because apparent intestinal disorders are consistently observed in such populations and they stimulate HF progression^170^. In patients with gastric cancer with coronary heart disease and HF, probiotic capsules containing *B. longum* enhanced the intestinal barrier and corrected intestinal microbial imbalance as well as HF, thereby promoting patient rehabilitation^170^.

Several clinical trials have reported the positive effects of bifidobacteria in HF patients. However, these clinical trials used probiotic combinations containing bifidobacteria rather than individual *Bifidobacterium* strains alone, which blurred the role of bifidobacteria to some extent. Preclinical and clinical studies should be performed to elucidate on the independent beneficial effects of bifidobacteria in HF patients.

### Prospects

This review has elucidated the critical role of bifidobacteria in the prevention of CVD development. On the one hand, bifidobacteria prevented damage to cardiac functions through the maintenance of intestinal barrier function and its antioxidant and immunomodulatory effects directly. On the other hand, bifidobacteria can also indirectly reduce the cardiac burden of obesity, HLP, and T2DM by affecting hormone secretion, regulating metabolism, assimilating cholesterol, and improving IR. In addition, bifidobacteria have found extensive application and demonstrated promising progress in various classic CVDs, such as HP, AS, MI, and HF. Indeed, some unique metabolic functions of *Bifidobacterium* may play a non-negligible role in cardiovascular disease, such as the metabolism of tryptophan, the production of its indole derivatives, and the regulation of peripheral serotonin, but the lack of evidence leads to the need for more attention and in-depth exploration.

It is important to recognize that advances in the field of molecular biology and genetic engineering techniques will pave the way for in-depth studies into all probiotics, including bifidobacteria. In recent years, several mutagenesis methods have been introduced in the field of bifidobacteria, including homologous recombination systems with non-replicating plasmids or temperature-sensitive plasmids, random rotor-based mutagenesis systems, and inducible plasmid self-destruction-assisted systems^171–174^. Although these strategies have some shortcomings, there are efforts to circumvent these problems through strategies, such as the development of endogenous CRISPR-Cas systems^171^. On the one hand, recent advances in the field of molecular biology and genetic engineering techniques have facilitated a deeper understanding of bifidobacteria-host interactions^175^. For example, by analyzing the genome sequence and in vivo transcriptome studies of *Bifidobacterium breve*, O’Connell^176^ identified a key site that influences its colonization of the mouse host intestine and highlighted that the ability of different strains to colonize the intestine is highly correlated with their ability to utilize carbohydrates. Furthermore, genetic engineering holds profound significance for forthcoming therapeutic advancements and industrial applications involving bifidobacteria. By harnessing sophisticated genetic engineering methods, the potential exists to enhance stress resilience, engineer targeted delivery systems to combat pathogens, eradicate antibiotic resistance, achieve luciferase labeling, and even generate entirely novel transgenic bifidobacterial strains that surpass wild-type counterparts in both efficacy and safety^177^, which can help develop novel therapeutic approaches and facilitate further clinical optimization.

In summary, addressing and controlling the bifidobacterial composition holds promise for advancing innovative therapeutic strategies in the context of CVD. The manipulation of the *Bifidobacterium* genome via genetic engineering to create pertinent foods and medications is anticipated to pave the way for safer and more efficient utilization of *Bifidobacterium* in the future, augmenting its potential as a treatment for CVD in human populations.
